# Time Course Analysis Reveals Gene-Specific Transcript and Protein Kinetics of Adaptation to Short-Term Aerobic Exercise Training in Human Skeletal Muscle

**DOI:** 10.1371/journal.pone.0074098

**Published:** 2013-09-12

**Authors:** Brendan Egan, Paul L. O’Connor, Juleen R. Zierath, Donal J. O’Gorman

**Affiliations:** 1 Institute for Sport and Health, School of Public Health, Physiotherapy, and Population Sciences, University College Dublin, Dublin, Ireland; 2 Department of Molecular Medicine and Surgery, Section of Integrative Physiology, Karolinska Institute, Stockholm, Sweden; 3 School of Health and Human Performance, Dublin City University, Dublin, Ireland; 4 Department of Physiology and Pharmacology, Karolinska Institute, Stockholm, Sweden; 5 Centre for Preventive Medicine, Dublin City University, Dublin, Ireland; National Institute of Child Health and Human Development, United States of America

## Abstract

Repeated bouts of episodic myofibrillar contraction associated with exercise training are potent stimuli for physiological adaptation. However, the time course of adaptation and the continuity between alterations in mRNA expression and protein content are not well described in human skeletal muscle. Eight healthy, sedentary males cycled for 60 min at 80% of peak oxygen consumption (VO_2peak_) each day for fourteen consecutive days, resulting in an increase in VO_2peak_ of 17.5±3.8%. Skeletal muscle biopsies were taken at baseline, and on the morning following (+16 h after exercise) the first, third, seventh, tenth and fourteenth training sessions. Markers of mitochondrial adaptation (Cyt c and COXIV expression, and citrate synthase activity) were increased within the first week of training, but the mtDNA/nDNA ratio was unchanged by two weeks of training. Accumulation of PGC-1α and ERRα protein during training suggests a regulatory role for these factors in adaptations of mitochondrial and metabolic gene expression. A subset of genes were transiently increased after one training session, but returned to baseline levels thereafter, which is supportive of the concept of transcriptional capacity being particularly sensitive to the onset of a new level of contractile activity. Thus, gene-specific temporal patterns of induction of mRNA expression and protein content are described. Our results illustrate the phenomenology of skeletal muscle plasticity and support the notion that transcript level adjustments, coupled to accumulation of encoded protein, underlie the modulation of skeletal muscle metabolism and phenotype by regular exercise.

## Introduction

The phenomenon of skeletal muscle plasticity is illustrated by the remodeling of muscle’s structure and functional make-up, as seen in muscular force, endurance and contractile velocity, as a result of alterations in functional demand [1], [2]. These adaptations in skeletal muscle to exercise training are widely-hypothesized to result from the gradual alteration of protein content consequent to repeated, but transient, alterations in transcript abundance induced by individual, acute bouts of exercise [3], [4]. A single bout of aerobic exercise increases the relative transcript abundance of many genes including myogenic factors, genes of carbohydrate metabolism, lipid mobilization, transport and oxidation, mitochondrial metabolism and oxidative phosphorylation, and transcriptional regulators of mitochondrial biogenesis [5]–[8]. Transcript induction is transient, but generally several-fold above resting values, peaking 3–12 h after cessation of exercise, and returning to basal levels within 24 hours [5], [9]. These transient increments are generally associated with a same directional change in the encoded protein to a new ‘relative steady-state’ level [3]. Continuity between the induced transcript and a change in protein content has been reported [10], but is not always present in exercise models [11], [12]. Short-term exercise training (e.g. less than 14 days) results in qualitatively similar adaptations to those observed after more prolonged training [13]–[15]. This is an attractive model of rapid phenotypic adaptation as the half-life for an increase in maximal aerobic capacity is estimated at eight to ten exercise training sessions [16], [17]. In addition, molecular events underlying training adaptations in this time frame occur at the level of the mitochondrion [15], and metabolic [13], intracellular signaling [18], and transcriptional [5] responses.

Recent progress in transgenic mouse models, combined with reductionist cell culture experiments *in vitro*, have added new perspectives on the regulation of skeletal muscle plasticity at a transcriptional level [3], [19]. In skeletal muscle, an increasingly well-defined network of transcription factors and co-regulator proteins has emerged as coordinators of adaptation to metabolic stress, including peroxisome proliferator-activated receptor (PPAR) γ coactivator 1α (PGC-1α), the transcriptional co-activator that is considered to be a “master regulator” of transcriptional processes through its co-regulation of various transcription factors [20]–[22]. This network provides a critical level of molecular control over metabolic and mitochondrial adaptation, illustrated by their ability to alter the expression of key enzymes in carbohydrate and lipid metabolism, and of the coordination of mitochondrial biogenesis [21], [22]. In general, a paucity of information exists regarding their involvement in a human exercise context beyond a transient, but robust, increase in mRNA expression after a single bout of exercise [5], [6], [8], [23], [24]. Therefore, whereas training-induced alterations in biochemical regulation in skeletal muscle and the metabolic consequences during subsequent exercise are well-described [1], less is known about the coordinated expression of mRNA and protein abundance induced by acute exercise, the continuity with exercise training, and the expression of regulatory factors accounting for the phenotypic change.

We have recently reported that extensive remodeling of the human skeletal muscle mitochondrial proteome is achieved by short-term aerobic exercise training [25]. The purpose of the present study was to employ a time course analysis in order to explore the molecular basis for this response by investigating the expression profile of transcriptional regulators and metabolic genes in response to the same training stimulus. Additionally, we examined the relationship between training-induced alterations in transcript abundance and the translated protein of genes known to be responsive to a *single* bout of exercise. Our results support recent proposals that skeletal muscle transcriptional capacity is particularly sensitive to a single bout of exercise [10], and that transcript level adjustments precede changes in the encoded protein product as part of molecular mechanisms that underlie the modulation of skeletal muscle phenotype by regular exercise.

## Methodology

### Participants and Ethical Approval

Eight healthy, sedentary males (23±2 yr, 1.79±0.03 m, 75.3±3.0 kg, 23.6±0.9 kg·m^−2^, 13.3±2.2% body fat), volunteered to participate in the study as previously described [25]. Participants were physically inactive (defined as partaking in less than one day of at least 30 minutes of moderate physical activity *per week)* [26] for at least six months prior to induction into the study and each underwent a thorough medical screening and provided written informed consent prior to participation. All experimental procedures were approved by the Dublin City University Research Ethics Committee, and conducted according to the principles expressed in the *Declaration of Helsinki* (http://www.wma.net/en/30publications/10policies/b3/).

### Experimental Design

**Figure 1:**
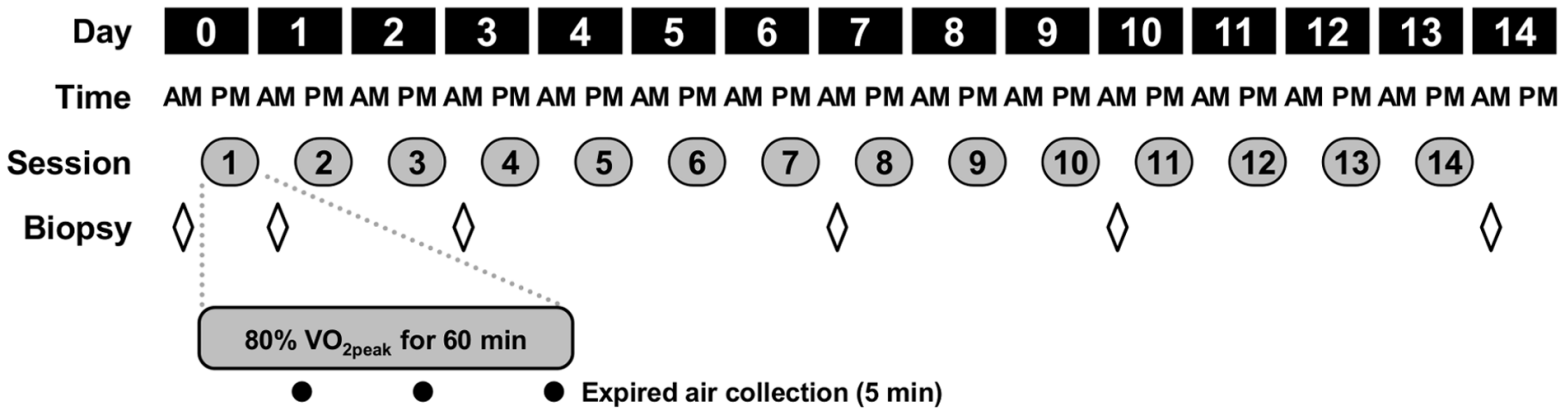
Study design for time course analysis of exercise training-induced changes in skeletal muscle gene expression. Each subject performed fourteen consecutive days of exercise training on a stationary cycle ergometer with one session each day for 60-training 80%VO_2peak_. Skeletal muscle biopsies were taken at baseline (day 0), and on the morning following the first, third, seventh, tenth and fourteenth training sessions. In order to monitor exercise intensity, oxygen uptake was measured for 5 min at three time points (15–20 min, 35–40 min and 55–60 min) during each training session.

The experimental design consisted of participants cycling for 60 min per exercise training session at ∼80% peak oxygen uptake (VO_2peak_) on fourteen consecutive days. Muscle biopsies were taken on the morning prior to the first training session, and five other mornings during the training programme (Figure 1). A test for peak oxygen uptake (VO_2peak_) was performed 72 h after the last training session to measure training-induced changes in whole-body maximal aerobic capacity.

### Exercise Testing

VO_2peak_ (2.81±0.15 L·min^−1^) was determined by indirect calorimetry (Vmax 29C, SensorMedics, Yorba Linda, CA) using an incremental protocol on an electronically-braked stationary cycle ergometer (Ergoline 900, SensorMedics). Briefly, participants began cycling at 80 W for five minutes of warm-up, after which the power output was increased by 40 W every two minutes thereafter until volitional fatigue. Oxygen uptake was considered to have peaked if two of the following criteria were met: (i) a leveling off of VO_2_ with increasing power output (increase of less than 2 mL·kg^−1^·min^−1^), (ii) a HR within 10 beats of the age predicted HR_max_ (220 bpm – age in years), (iii) a RER greater than 1.10. Expired air was collected continuously throughout exercise and analyzed using the Vmax 29C system. Heart rate was continuously monitored during exercise by telemetry (Polar Vantage NVTM; Polar, Port Washington, NY). Between four and seven days after the VO_2peak_ test, each participant returned to the laboratory to perform a test to verify the power output required to elicit the requisite VO_2peak_ for each training session, i.e. 80% VO_2peak_. The power output required to elicit a given percentage of VO_2peak_ was interpolated based on the linear relationship between oxygen uptake (*y*-axis) and power output (*x*-axis). For the given percentage of VO_2peak_, the corresponding power output is estimated by solving for *x* using the linear function, *y = mx+c*, where *y* is VO_2_ (L·min^−1^), *x* is power output (W), *m* is the slope of the relationship between VO_2_ and power output, and *c* is the y-axis intercept. Seven days after the test to verify the power output required for training, the training programme commenced.

### Exercise Training Programme

The exercise training programme consisted of fourteen exercise training sessions performed on fourteen consecutive days, beginning on day 0 (Figure 1). Each training session was performed under supervision in the Human Performance Laboratory at Dublin City University. Each training session was 60 min in duration at an intensity equivalent to 80% of pre-training VO_2peak_. Plain water was allowed for consumption *ad libitum* throughout each session. Training sessions for each individual took place at the same time of day ±1 h to preclude any influence of circadian variation on the response to individual training sessions. Expired air was collected for 5 min on three occasions during each session; 15–20 min, 35–40 min, 55–60 min. From this, VO_2_ was monitored as the measure of exercise intensity, and the power output was adjusted accordingly to elicit the target exercise intensity. Values for VO_2_, VCO_2_, RER, V_E (STPD)_, and F_E_O_2_ were recorded from the expired air analysis using 60 s averages and used *post-hoc* to calculate the rate of energy expenditure [27]. Each 5 min sampling period was assumed to be representative of the average energy expenditure for the preceding 15 min and hence, cumulative energy expenditure per session was estimated based on the summation of these three 20 min periods. The target oxygen uptake was increased by 10% after seven sessions in an attempt to ensure that the absolute training stimulus was increased commensurate with the expected increase in VO_2peak_. Using a similar training protocol, an approximately 10% increase in VO_2peak_ was observed after seven to ten days of cycle ergometer training [15].

### Skeletal Muscle Biopsies

Skeletal muscle specimens were taken by muscle biopsy from the *m. vastus lateralis* under local anesthesia. An area of skin, subcutaneous tissue, and fascia was anaesthetized with 2% w/v Lidocaine HCl and a small (0.5 cm) incision made. The biopsy needle was inserted into the muscle and approximately 200 mg of tissue removed using the percutaneous muscle biopsy technique with suction applied [28]. Muscle samples were snap-frozen in liquid nitrogen and stored at −80°C until analysis. Six muscle biopsies were taken during the training programme (Figure 1). On the morning of day 0, participants reported to the Metabolic Physiology Research Unit after an overnight (>8 h) fast and were requested to rest quietly in a supine position for approximately 15 min. A resting muscle biopsy was taken (#1, Baseline/day 0). On the morning following the first exercise session, participants reported again after an overnight fast and rested quietly as before. A second muscle biopsy was taken. The biopsy was taken 16 h after the cessation of the last exercise training session. This pattern was repeated for remaining biopsies, which were taken on the mornings following (+16 h) the third, seventh, tenth and fourteenth training sessions. A fresh incision was made for each biopsy and was 3 cm proximal or distal to a previous biopsy site.

### Dietary Control

For the three days prior to day 0, participants were required to keep a record of all food and fluid consumed. Participants were then asked to repeat this pattern of intake throughout the training phase to reduce the variability in metabolic responses that may have been due to day-to-day variation in dietary intake. Participants were weighed to the nearest 0.1 kg in minimal clothing prior to and immediately after each training session to determine fluid loss induced by each session. Each participant was then provided with a volume of plain water equivalent to 150% of the difference in body mass i.e. for every kg of body mass lost during the session, 1.5 L of water was provided. At this time, each participant was given two cereal bars (Nutrigrain®, Kellogg’s, UK) for consumption with the bolus of fluid. This provided 50 g of CHO, 3 g of protein and 7 g of fat immediately after each session.

### Quantitative Real-time Polymerase Chain Reaction (qPCR)

Total RNA was isolated from approximately 20 mg of crude muscle using TRIzol reagent (Sigma-Aldrich, UK) as per the manufacturer’s instructions. Total RNA concentration was quantified spectrophotometrically at an absorbance of 260 nm (NanoDrop ND-1000 Spectrophotometer, ThermoFisher Scientific, Waltham, MA, USA). The integrity and purity of each RNA sample was verified by gel electrophoresis (RNA integrity number, RIN>7; RNA 6000 Nano Lab Chip and 2100 Bioanalyzer, Agilent Technologies, Palo Alto, CA, USA) and by measuring the spectrophotometric A260/A280 (>1.8) and A260/A230 (>1.5) ratios. RNA (2 µg) was reverse transcribed to cDNA using the Reverse Transcription System (Promega, Madison, WI, USA) primed with oligo-dT_(15)_ as per the manufacturer’s instructions. The cDNA template was stored at −20°C until subsequent analysis. Relative mRNA expression (20 ng cDNA template per reaction) was determined using quantitative real-time PCR (ABI Prism 7500, Applied Biosystems, Foster City, CA, USA) using Assay-On-Demand® primer pairs and probes (P/N 4331182, Taqman® Gene Expression Assays, Applied Biosystems) and Taqman® Universal PCR MasterMix (Applied Biosystems). Assay IDs for specific mRNA targets are listed in Table S1 in File S1. GAPDH mRNA expression (4333764F, Applied Biosystems) was stable across all time points during training and used as the housekeeping gene to which target mRNA expression was normalized. The average threshold cycle number (C_T_) values of the unknown samples were converted to relative expression data using an appropriate standard curve of serial dilutions of pooled cDNA.

### Immunoblot Analysis

**Figure 2:**
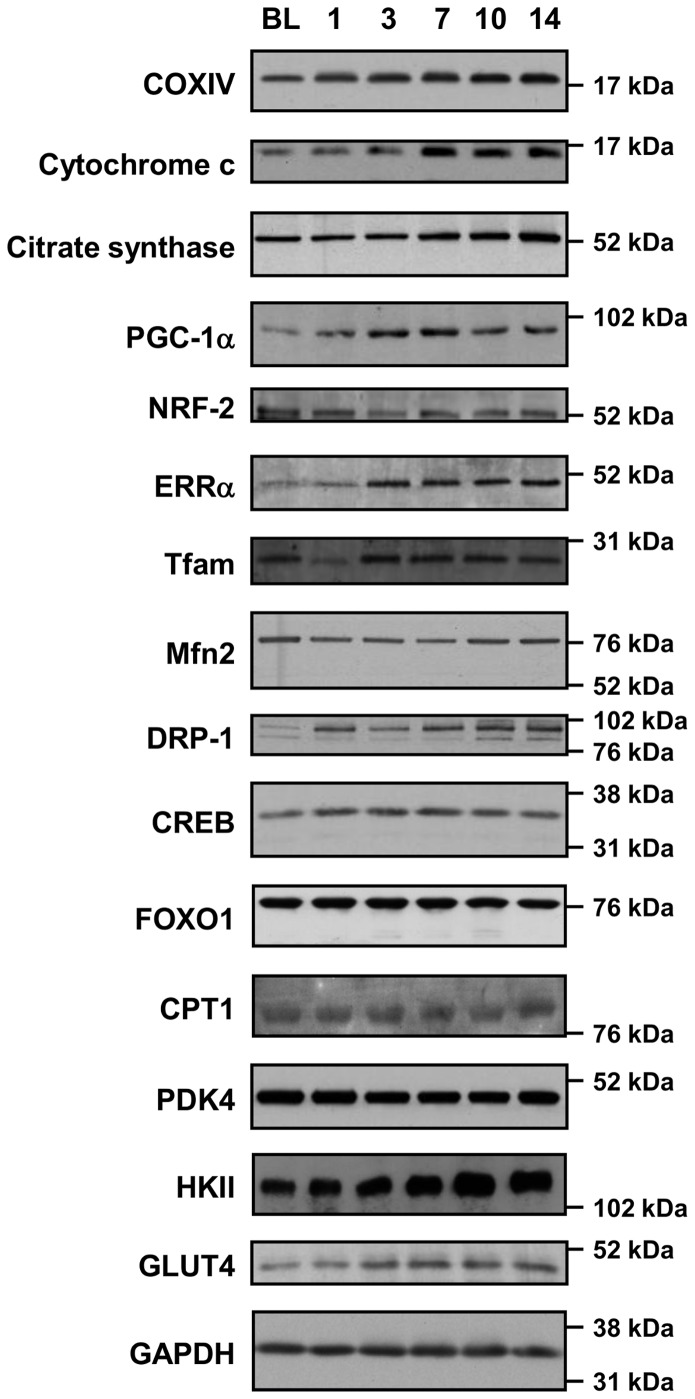
Representative immunoblots for proteins of interest. Protein extracts from skeletal muscle biopsies prior to training (BL, baseline) and after 1, 3, 7, 10 and 14 days of aerobic exercise training were resolved by SDS-PAGE and subjected to immunoblot directed against the protein of interest indicated. Approximate location of molecular weight markers (RPN 800E; GE Healthcare/Amersham) is also indicated. See Table S1 in File S1 for full list of abbreviations.

Approximately 20 mg of crude muscle was freeze-dried, dissected free of blood and connective tissue, and homogenized in 50 µl per mg freeze-dried muscle of ice-cold homogenization buffer (20 mM Tris [pH 7.8], 137 mM NaCl, 2.7 mM KCl, 1 mM MgCl_2_, 1% Triton X-100, 10% [wt/vol] glycerol, 10 mM NaF, 1 mM EDTA, 5 mM sodium pyrophosphate, 0.5 mM Na_3_VO_4_, 1 µg/ml leupeptin, 0.2 mM phenylmethyl sulfonyl fluoride, 1 µg/ml aprotinin, 1 mM dithiothreitol, 1 mM benzamidine, 1 µM microcystin) using a motorized pestle. Homogenates were rotated end-over-end for 60 min at 4°C, centrifuged (12,000 *g* for 15 min at 4°C), and the protein content of the supernatant was determined by a commercially available detergent-compatible colorimetric assay (Bio-Rad Laboratories, Hercules, CA). An aliquot of muscle homogenate (20 µg protein) was mixed with Laemmli buffer (20% glycerol, 62.5 mM Tris-HCl, 2% SDS, 0.00125% bromophenol blue, 2% β-mercaptoethanol) and separated in one-dimension by SDS-PAGE on Criterion XT pre-cast 4–12% gradient Bis-Tris gels (345-0124; Bio-Rad), before transfer to polyvinylidene difluoride (PVDF) membranes using the Criterion Cell and Blotter systems (Bio-Rad) as per the manufacturer’s instructions. Briefly, electrophoresis was performed in pre-chilled 1X XT MES running buffer (161-0789; BioRad), for approximately 90 min at 100 V, with cooling on ice, until the dye-front reached 1 cm from the base of the gel. Proteins were immobilized by transfer to PVDF membranes (Immobilon P, IPVH00010; Millipore, Billerica, MA, USA) at 200 V for 75 min in pre-chilled transfer buffer (25 mM Tris, 192 mM glycine, 10% methanol, 0.1% SDS) with cooling on ice. Following transfer, non-specific protein binding was blocked in a 7.5% milk/2.5% BSA/TBS-t (10 mM Tris pH 7.5, 100 mM NaCl, 0.4% Tween20) for 2 h at room temperature. Membranes were incubated overnight with primary antibodies directed towards proteins of interest. Details of each primary antibody are provided in Table S1 in File S1. Membranes were washed in TBS-t and incubated with appropriate secondary HRP-conjugated antibodies (Bio-Rad), visualized by ECL (GE Healthcare/Amersham, Uppsala, Sweden) and quantified by densitometry (GS800 Calibrated Imaging Densitometer, Bio-Rad). Representative blots are included where appropriate. Consistency of protein loading per lane was assessed by reversible Ponceau staining [29], and confirmed by immunoblot for GAPDH (sc-25778; Santa Cruz Biotechnology, Santa Cruz, CA, USA). Representative immunoblots for proteins of interest are shown in Figure 2.

### Quantification of Mitochondrial DNA-to-nuclear DNA (mtDNA/nDNA) Ratio

Approximately 10 mg of crude muscle was freeze-dried, dissected free of blood and connective tissue, after which genomic DNA was extracted using DNeasy Blood & Tissue columns (Qiagen, Valencia, CA, USA) according to the manufacturer’s instruction. Total DNA concentration was quantified spectrophotometrically (NanoDrop). mtDNA/nDNA ratio was determined by quantifying expression of mitochondrial DNA (non-coding) and the nuclear DNA (non-coding intra-*Alu*Yb8) by real-time PCR (ABI Prism 7500, Applied Biosystems) as previously described [30]. Purified DNA (5 µL at 5 ng µL^−1^) was amplified in a 25 µL PCR reaction containing SYBR Green Master Mix (Applied Biosystems) and 100 nM of each primer. qPCR for the detection of mtDNA and nDNA was performed as two separate reactions, but within the same plate run for each sample. All samples were run in duplicate for each DNA target. The primers were human-specific and designed to target mtDNA (forward: AATATTAAACACAAACTACCACCTACCT; reverse: TGGTTCTCAGGGTTTGTTATAA) or nDNA (forward: CTTGCAGTGAGCCGAGATT; reverse: GAGACGGAGTCTCGCTCTGTC). Serial dilutions of pooled DNA were run for each DNA target to generate a standard curve allowing for assessment of qPCR amplification efficiency and quantification of mtDNA and nDNA in relative copy number based on respective C_T_ values.

### Citrate Synthase Activity Assay

Maximal citrate synthase activity was determined in whole muscle homogenates using a commercially available assay kit (CS0720; Sigma-Aldrich, Poole, UK), with absorbance read at 412 nM (SpectraMax; Molecular Devices, Sunnyvale, USA).

### Statistical Analysis

Data were evaluated using the SigmaStat for Windows v3.11 software package (Systat Software, Inc, San Jose, CA, USA). One-way repeated measures ANOVA was used determine the effect of exercise training on variables of interest with serial measurements. *Post-hoc* pair-wise comparisons using Fisher’s least significant difference (LSD) were performed when a significant F-score was detected. A paired t-test was used to determine the differences between variables for pre- to post-training comparison, i.e. VO_2peak_ scores and changes in body mass. The significance level was set at α = 0.05 for all statistical tests. All data are reported as mean ± SEM.

## Results

### Exercise Training Log and Improvement in Aerobic Capacity

**Figure 3:**
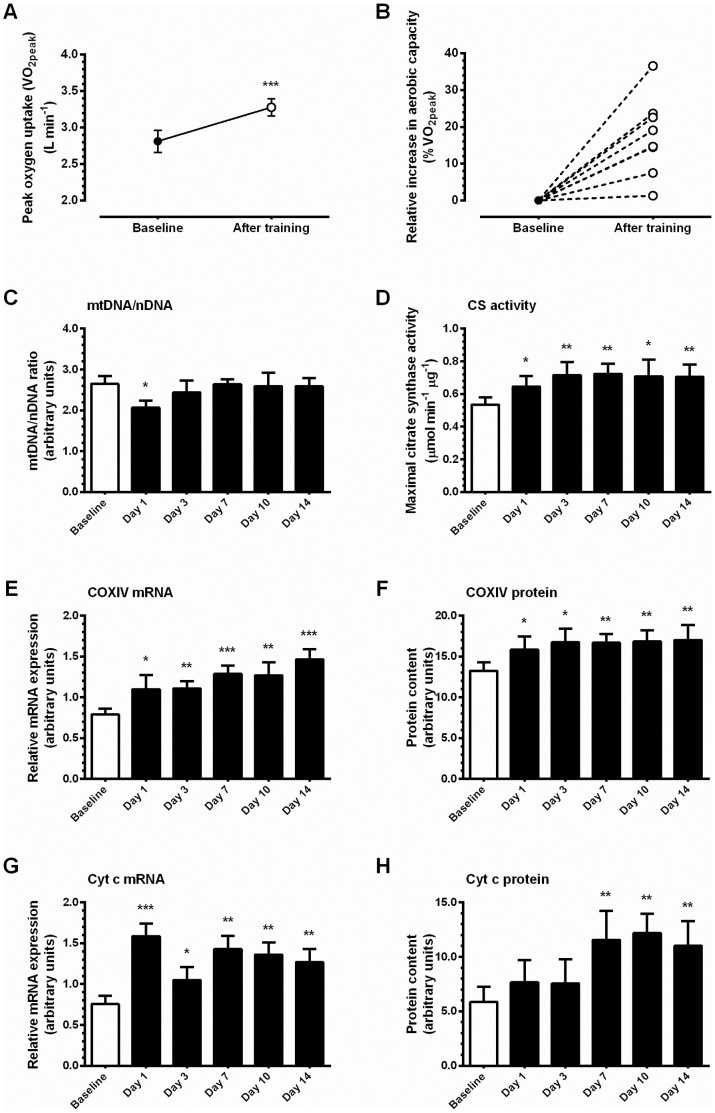
Effect of exercise training on maximal oxygen uptake (*A&B*), and markers of mitochondrial adaptation (*C-H*). Aerobic capacity was determined prior to training (Baseline) and 72 h after the last training session (After training). **A,** average group improvement in aerobic capacity in response to exercise training. **B,** individual changes in aerobic capacity expressed as percentage increase from Baseline. **C,** relative copy number of mtDNA and nDNA was determined by qPCR directed toward non-coding DNA fragments in the mitochondrial and nuclear genomes. **D,** maximal enzymatic activity of citrate synthase. **E,** relative mRNA expression of COXIV. **F,** protein content of COXIV. **G,** relative mRNA expression of cytochrome c. **H,** protein content of cytochrome c. mRNA expression was normalized to the housekeeping gene GAPDH. Values are mean ± SEM, n = 7–8. * trend for a significant difference compared to Baseline (P<0.1), ** significantly different from Baseline (P<0.05), *** significantly different from Baseline (P<0.005).

Each participant completed his full complement of fourteen days of exercise training as described, indicating 100% compliance. The specifics of exercise intensity and energy expenditure are described in the training log (Table S2 in File S1). Exercise training resulted in 17.8±3.5% increase in VO_2peak_ compared to pre-training values (P = 0.002, Figure 3A), with individual improvements ranging from 1.3% to 36.6% (Figure 3B). Average exercise intensity of the fourteen training sessions was 80.5±1.9% of the pre-training VO_2peak_, which represents 68.8±2.3% of the post-training VO_2peak_. The average change in body mass from baseline to day 14 was −0.4±0.3 kg (P = 0.209).

### Mitochondrial Adaptation to Short-term Training

#### mtDNA/nDNA ratio

After one training session, the mtDNA/nDNA ratio tended to be decreased (−22%, P = 0.059). This decrease was observed in all but one of the participants. Analysis of these seven participants indicated a 28% decrease in the mtDNA/nDNA ratio (P = 0.008). The robustness of this finding was confirmed (data not shown) using a second primer set targeting coding regions of mtDNA (mitochondria-encoded NADH dehydrogenase subunit 1) and nDNA (lipoprotein lipase) as previously described [31].

#### Citrate synthase activity

Maximal activity of citrate synthase (CS), a classic marker of skeletal muscle mitochondrial adaptation to exercise training [1], tended to be increased after one exercise session, and was ∼35% greater at day 3 compared to pre-training values (P<0.05), where it remained for the remainder of the training phase (Figure 3D). Relative mRNA expression of CS was also elevated from day 3 to 14 (P<0.05; Table 1), although the increases in maximal CS enzymatic activity preceded an increase in CS protein, which was only detectable from day 7 onwards (Table 1).

**Table 1:** Training-induced alterations in gene expression of selected transcriptional regulators, and metabolic and mitochondrial genes.

	Baseline	Day 1	Day 3	Day 7	Day 10	Day 14
*Transcriptional regulators*						
RIP140 mRNA	1.00±0.12	2.05±0.17**	1.36±0.06*	1.14±0.06	1.16±0.10	1.11±0.06
NRF-1 mRNA	1.00±0.09	1.51±0.18**	1.26±0.12*	1.18±0.09*	1.15±0.09	1.28±0.12
Mfn1 mRNA	1.00±0.06	1.65±0.17**	1.19±0.07*	1.31±0.07*	1.36±0.05*	1.25±0.09*
Mfn2 mRNA	1.00±0.11	0.83±0.19	1.95±0.17**	1.53±0.14**	1.34±0.19*	1.61±0.17**
PPARδ mRNA	1.00±0.08	1.68±0.30*	0.93±0.09	0.93±0.08	0.87±0.05	0.94±0.07
CREB mRNA	1.00±0.07	1.43±0.16**	1.26±0.08**	1.25±0.05**	1.25±0.07**	1.30±0.07**
CREB protein	1.00±0.15	1.27±0.09*	1.08±0.15	1.36±0.11**	1.28±0.09**	1.03±0.12
FOXO1 mRNA	1.00±0.19	2.02±0.23**	1.28±0.13	1.08±0.12	1.22±0.17	1.19±0.15
FOXO1 protein	1.00±0.09	1.10±0.10	1.04±0.18	0.98±0.18	1.10±0.09	1.13±0.14
*Metabolic and mitochondrial genes*						
CPT1 mRNA	1.00±0.13	0.85±0.21	2.01±0.14**	1.54±0.11**	1.54±0.17**	1.75±0.12**
CPT1 protein	1.00±0.16	1.00±0.24	1.08±0.17	1.20±0.12	1.12±0.25	1.39±0.29*
CS mRNA	1.00±0.10	1.17±0.09	1.76±0.08**	1.58±0.10**	1.25±0.07**	1.53±0.09**
CS protein	1.00±0.11	1.03±0.12	1.09±0.12	1.22±0.10*	1.45±0.06***	1.60±0.14**

See Table S1 in File S1 for abbreviations. mRNA expression was normalized to the housekeeping gene GAPDH. Values are mean ± SEM, n = 7–8. *trend for a significant difference compared to Baseline (*P*<0.1), **significantly different from Baseline (*P*<0.05), ***significantly different from Baseline (*P*<0.005).

#### Mitochondrial electron transport chain

Exercise training increased mRNA and protein expression of cytochrome c oxidase subunit IV (COXIV) and cytochrome c (Cyt c), two components of the mitochondrial electron transport chain (Figure 3E–H). COXIV mRNA expression increased progressively throughout the training period, reaching values 1.9-fold greater than baseline expression (*P*<0.05; Figure 3E), whereas protein expression was elevated after one session (20%), and remained elevated thereafter (∼30%, P<0.05; Figure 3F). Cyt c mRNA expression increased after one session (2.1-fold), and remained elevated (P<0.05) throughout training despite a tendency to decline progressively from day 7 to 14 (Figure 2G). However, Cyt c protein content was unchanged at either day 1 or 3, but was ∼2-fold greater than baseline on days 7, 10 and 14 (P<0.05; Figure 3H).

### PGC-1 Family of Coactivators and Related Coregulators

**Figure 4:**
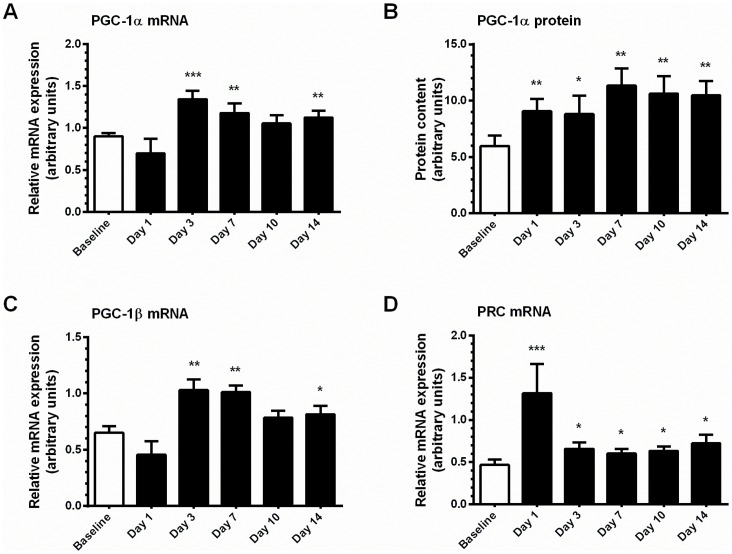
Training-induced alterations in gene expression of members of the PGC-1 family of transcriptional coactivators. **A**, relative mRNA expression of PGC-1α. **B**, protein content of PGC-1α. **C**, relative mRNA expression of PGC-1β. **D**, relative mRNA expression of PRC. mRNA expression was normalized to the housekeeping gene GAPDH. Values are mean ± SEM, n = 7–8. * trend for a significant difference compared to Baseline (P<0.1), ** significantly different from Baseline (P<0.05), *** significantly different from Baseline (P<0.005).

Expression of PGC-1α mRNA increased above pre-training values from day 3 onwards (48%, P<0.05), and declined thereafter but remained 25% higher than baseline at day 14 (P<0.05; Figure 4A). The corresponding protein content was increased (52%, P<0.05) after one session of training, and remained elevated throughout the training programme (∼75–90%, P<0.05; Figure 4B). Alterations in PGC-1β mRNA expression displayed a similar pattern to PGC-1α mRNA (Figure 4C), whereas mRNA expression of PGC-1-related coactivator (PRC), a PGC-1 family member linked to the regulation of mitochondrial adaptation, was markedly increased after one exercise session (2.8-fold, P<0.05), and tended to remain elevated throughout training (Figure 4D). We were unable to access reliable antibodies for protein analysis for either PGC-1β or PRC. Relative mRNA expression of the transcriptional corepressor nuclear receptor-interacting protein 1 (RIP140) and PPARδ, which acts as a positive regulator of oxidative phenotype in skeletal muscle, were significantly increased after one bout of exercise (2.1- and 1.7-fold, respectively, P<0.05), and RIP140 remained 50% above baseline expression at day 3 (P<0.05), but both targets returned to baseline values for the remainder of the study (Table 1).

### Transcriptional Regulators of Mitochondrial and Metabolic Gene Expression

**Figure 5:**
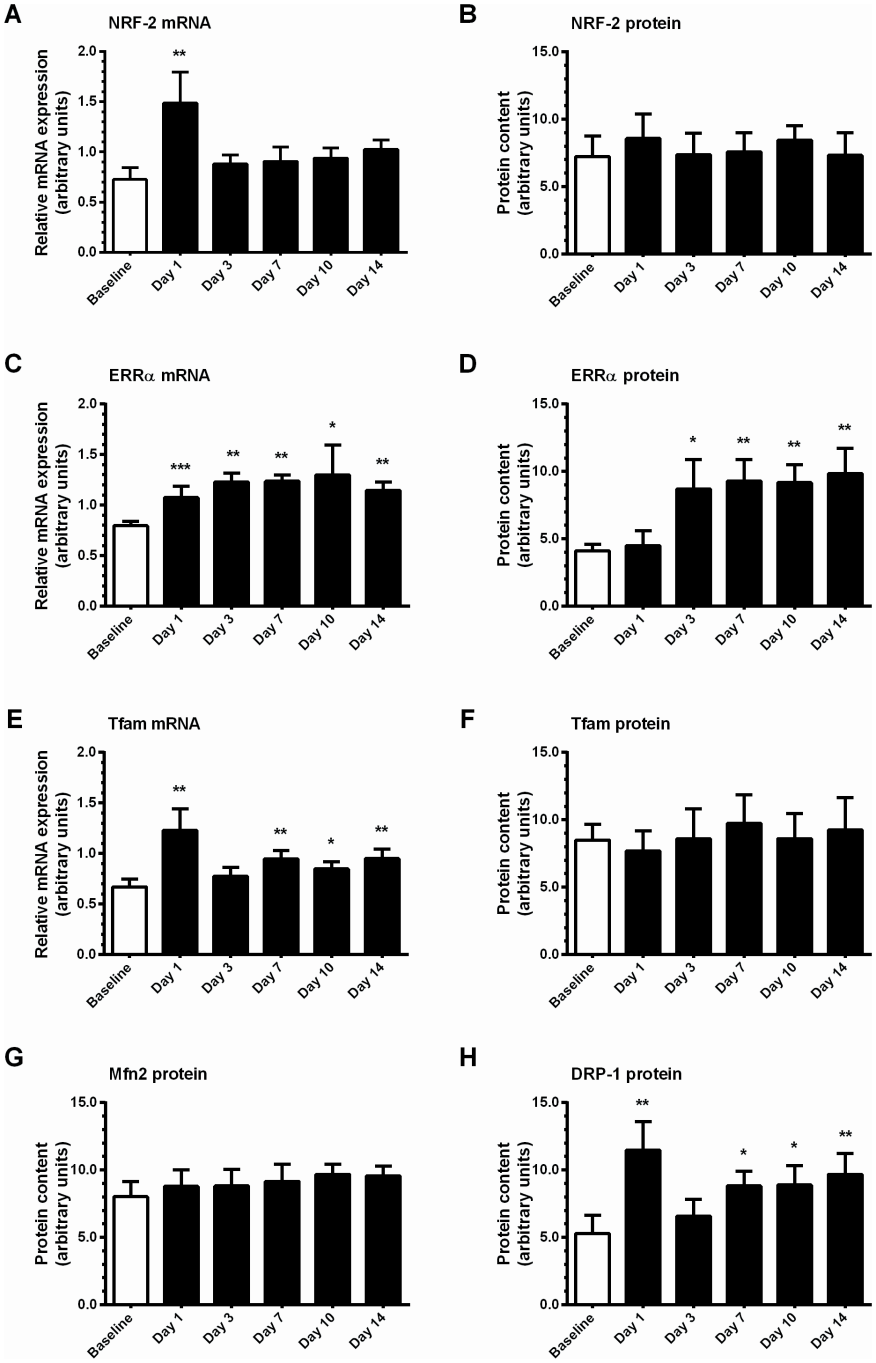
Training-induced alterations in the expression of selected transcriptional regulators of skeletal muscle gene expression. **A**, relative mRNA expression of NRF-2. **B**, protein content of NRF-2. **C**, relative mRNA expression of ERRα. **D**, protein content of ERRα. **E**, relative mRNA expression of Tfam. **F**, protein content of Tfam. **G**, protein content of Mfn2. **H**, protein content of DRP-1. mRNA expression was normalized to the housekeeping gene GAPDH. Values are mean ± SEM, n = 7–8. * trend for a significant difference compared to Baseline (P<0.1), ** significantly different from Baseline (P<0.05), *** significantly different from Baseline (P<0.005).

mRNA expression of nuclear respiratory factor-1 (NRF-1) (∼50%; Table 1) and NRF-2 (2-fold; Figure 5A), nuclear-encoded transcription factors linked to the transcriptional control of many mitochondrial genes, was elevated after the first exercise training session, but returned to values similar to pre-training values thereafter. No change in NRF-2 protein content was detected (Figure 5B), whereas we were unable to obtain reliable immunoblots for NRF-1 protein. Similar patterns to NRF-2 were observed for mitochondrial transcription factor A (Tfam), a nuclear-encoded transcription factor essential for the replication, maintenance, and transcription of mtDNA, with elevated mRNA expression after the first training session, and no change in protein content throughout training (Figure 5E&F). However, Tfam mRNA expression was elevated by ∼40% at day 7 and 14 (P<0.05). Expression of FOXO1, a forkhead transcription factor regulating expression of genes involved in energy metabolism and shifts in fuel selection, often in concert with estrogen-related receptor α (ERRα), showed a similar response as NRF-2, i.e. an mRNA induction after one session of training (P<0.05), which returned to baseline thereafter and no change in protein content during training (Table 1). mRNA expression of ERRα, a constitutively-active orphan nuclear receptor important for regulation of oxidative phosphorylation, fatty acid oxidation, mtDNA expression, and angiogenesis, was elevated from day 1 onwards (∼40–50%, P<0.05; Figure 5C), and a corresponding 2.1- to 2.4-fold elevation in protein content above the pre-training content was observed (P<0.05; Figure 5D). Mitofusins (Mfn) act as regulators of mitochondrial fusion, and Mfn1 mRNA expression was elevated by 65% (P<0.05) after the first training session, and tended to remain elevated thereafter, whereas Mfn2 mRNA was elevated 2-fold (P<0.05) over baseline values on day 3 and remained elevated for the remainder of the training programme (Table 1). However, no measurable change in Mfn2 protein was detected during training (Figure 5G). A biphasic pattern of induction of Dynamin-related protein 1 (DRP-1) protein, a key regulator of mitochondrial fission, was observed such that DRP-1 protein content was elevated ∼2.2-fold (P<0.05) in response to the first training session, but returned to pre-training levels by day 3 before increasing by ∼65–80% from day 7 onwards (Figure 5H). Cyclic-AMP response element binding protein (CREB) mRNA expression was elevated on day 1 and remained modestly (∼25–30%) but significantly (P<0.05) elevated for the remainder of training (Table 1). CREB protein content was increased (P<0.05) at days 1, 7 and 10 (∼25–35%), but returned to baseline values by day 14 (Table 1).

### Markers of Metabolic Adaptation

**Figure 6:**
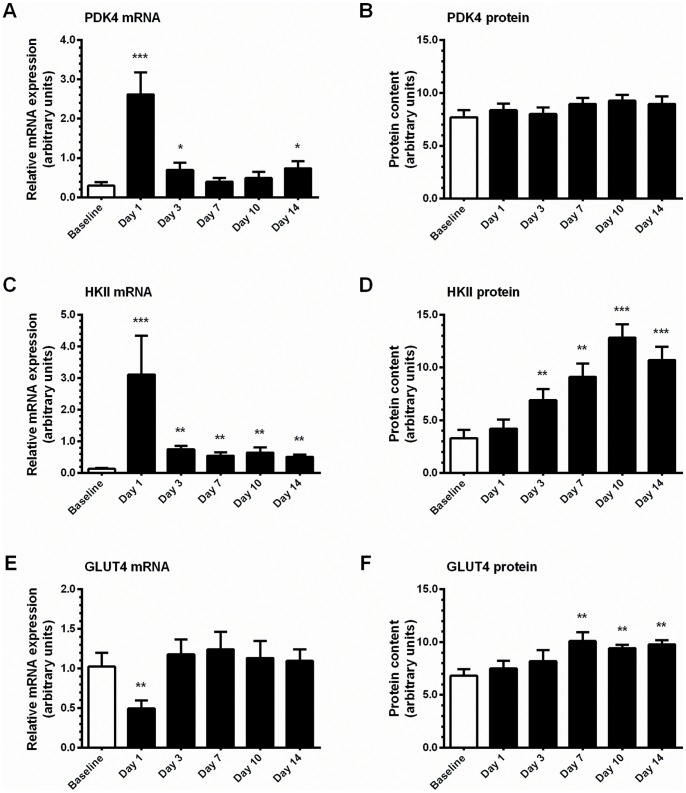
Training-induced alterations in gene expression of selected metabolic targets. **A,** relative mRNA expression of PDK4. **B,** protein content of PDK4. **C,** relative mRNA expression of HKII. **D,** protein content of HKII. **E,** relative mRNA expression of GLUT4. **F,** protein content of GLUT4. mRNA expression was normalized to the housekeeping gene GAPDH. Values are mean ± SEM, n = 7–8. * trend for a significant difference compared to Baseline (P<0.1), ** significantly different from Baseline (P<0.05), *** significantly different from Baseline (P<0.005).

Pyruvate dehydrogenase kinase 4 (PDK) 4 mRNA expression was markedly increased after the first training session (8.7-fold, P<0.05), and tended to be increased at day 3 and day 14 (Figure 6A). However, no change was noted in PDK4 protein content at any time point (Figure 6B). Hexokinase II (HKII) mRNA expression was elevated by more than 20-fold after the first bout of exercise, and remained 4- to 6-fold above pre-training expression levels for the remainder of the training program (P<0.05; Figure 6B). In turn, HKII protein content was elevated from day 3 onwards (P<0.05), peaking at 3.9-fold greater than baseline protein content on day 10 (Figure 6D). Expression of the facilitated glucose transporter, member 4 (GLUT4) was unaltered at mRNA level during training, but a marked decrease was observed after the first exercise bout (∼50%) after which expression levels returned to pre-training values (Figure 6E). In contrast, GLUT4 protein content increased after seven sessions of training, and remained approximately 40% higher (P<0.05) than baseline values for the remainder of the study (Figure 6F). Carnitine palmitoyltransferase 1 (CPT1) mRNA expression was increased by 1.5- to 2.0-fold from day 3 onwards (P<0.05), but was not reflected by a significant increase in protein content (Table 1).

## Discussion

In the present study, we report the temporal alterations in mRNA expression and protein content of key regulators of adaptation to exercise training in skeletal muscle of previously untrained men. Using short-term aerobic exercise training as a model of rapid phenotypic adaptation, we profiled gene-specific time courses of transcript and protein induction in response to repetitive contractile stimuli. Coordinated changes in PGC-1α and ERRα expression, temporally coincident with changes in classical markers of mitochondrial adaptation, support a role for these transcriptional regulators in the control of exercise-induced changes in skeletal muscle gene expression. A subset of genes that were transiently increased after one training session, but returned to baseline levels thereafter, is supportive of the concept of transcriptional capacity being particularly sensitive to the onset of a new level of contractile activity [10]. Moreover, we demonstrate that exercise training has divergent effects on temporal changes in mRNA expression and protein content for a variety of transcriptional regulators and their metabolic and mitochondrial targets.

### Mitochondrial Adaptations to Short-term Aerobic Exercise Training

Adaptive changes in mitochondrial number, density and metabolism are a well-established consequence of long-term aerobic exercise training [32], [33]. In the present study, we assessed the ratio of mitochondrial DNA to nuclear DNA as a marker of tissue concentration of mitochondria per cell, but this was unchanged after two weeks of aerobic exercise training. A higher mtDNA/nDNA ratio is reported in athletes coincident with higher maximal mitochondrial ATP synthesis rate and whole-body VO_2peak_ [31], [34], but recent work suggests that, in human skeletal muscle, mtDNA/nDNA ratio is a poor biomarker of mitochondrial content [35]. Instead CS activity and markers of OXPHOS activity such as complex V protein content are considered more informative [35]. In our intervention, we observed elevations in CS activity and progressive increases in Cyt c and COXIV expression during training, with changes evident within the first three to seven days of training. These observations, together with our recent work demonstrating remodeling of the mitochondrial proteome including complex V subunits ATP synthase α and β [25], are suggestive of mitochondrial biogenesis in response to as little as two weeks of exercise training.

Classically, it has been suggested that mitochondrial remodeling at the level of mitochondrial protein synthesis occurs with short-term exercise training but that *biogenesis* does not occur until later in the adaptive process [36], [37]. The approximate six weeks required to achieve a measurable increase mitochondrial content in response to aerobic training [36], [37] does not reflect early molecular events that lead to morphological changes. For instance, mitochondrial proteins turn over with a half-life of approximately seven days after the onset of contractile activity [38], whereas enzymatic activities increase after as little as two to three days of activity [10], [39]. Our findings are consistent with these observations, and indicate that changes in mRNA either precede or coincide with same-directional changes in mitochondrial enzyme activity and protein content (e.g. Figure 3, Table 1).

### Adaptive Responses of Transcriptional Regulators of Skeletal Muscle Gene Expression

A network of transcription factors and transcriptional coregulators including PGC-1α, NRF-1 and -2, ERRα, and Tfam are among the putative regulators of skeletal muscle gene expression [4], [21], [22], but their contribution to exercise training-induced adaptation in human skeletal muscle is not fully understood. Elevations in both mRNA and protein content for the transcriptional coactivator PGC-1α and the orphan nuclear receptor ERRα occurred in response to short-term training in the present study. PGC-1α and ERRα are established regulators of mitochondrial biogenesis and metabolic gene expression in skeletal muscle [40]–[42]. Altering the transcriptional activity of PGC-1α affects physiological responses that equip the cell to meet the energy demands of a changing environment, including augmentation of mitochondrial biogenesis, cellular respiration rates and substrate utilization [20]. ERRα regulates the expression of genes regulating oxidative phosphorylation [40], and fatty acid oxidation [41]. Both PGC-1α and ERRα mRNA expression are transiently increased during recovery from a single bout of exercise [8], [23], [24], and we observed an accumulation effect at protein level with repeated exercise bouts. To our knowledge, this is the first experiment to report changes in ERRα protein expression by exercise *training*.

Thus, the aforementioned changes in mitochondrial markers are likely to be linked to a PGC-1α/ERRα-associated regulation. In temporal terms, elevations in protein content of PGC-1α and ERRα after one and three training sessions respectively, coincided with the time course of increases in Cyt c, COXIV, and CS activity. PGC-1α- and ERRα-dependent regulation of COXIV has been established [43], and is mediated by coactivation of NRF-2 [44]. However, in the present study, it is not possible to ascertain whether training-induced increases in gene expression are due to PGC-1α coactivation of ERRα and NRFs, or a combination of other factors. In addition, we observed exercise-responsiveness for PGC-1β and PRC transcripts, each of which is associated with mitochondrial remodeling in skeletal muscle in concert with NRFs and ERRα [45], [46]. We cannot infer changes in protein content from our work, but PGC-1β protein is reported to increase during short-term high intensity interval training (HIIT) [10]. Thus, this pathway in association with other factors, such as NRFs, is likely to modulate exercise-induced skeletal muscle adaptation [4], [21], [22].

### Temporal mRNA-protein Kinetics of the Adaptive Response to Exercise Training

Several genes, including RIP140, NRF-1, NRF-2, PPARδ, Mfn-1 and FOXO1, showed elevated mRNA expression after the first training session, but in general returned to basal levels during the remainder of training. However, the relative mRNA expression of Mfn-2 and Tfam was elevated *throughout* training. Despite these divergent transcript changes, the protein content for NRF-2, Mfn-2, Tfam or FOXO1 was unaltered by training. This does not necessarily rule out a role for these factors in the regulation of skeletal muscle plasticity. A biphasic response such that an initially large transient elevation in transcript is followed laterally by only minor increments in mRNA and protein has been reported in a rodent model of chronic muscle activation [47]. However, unlike the stimulus provided by sustained chronic low-frequency electrical stimulation [2], exercise training represents an intermittent stimulus, and thus, changes in gene expression only reflect transient states between cycles of contractile activity, recovery and inactivity. Additionally, transcription factor DNA binding activity is controlled not by *total* protein content, but predominantly by posttranslational modifications of *existing* protein [48]. Therefore, basal content of these factors may be sufficient to coordinate exercise-induced adaptive processes, or alternatively, modifications in protein content may occur later than the time course investigated. As a case in point, whereas changes in relative mRNA expression of CS preceded changes in CS protein (day 7 onwards), maximal *enzyme* activity of CS increased within the first three days of training.

The kinetics of mRNA and protein induction are a function of the relationships between basal expression, the activation of transcription, and the turnover rate of a given mRNA and protein [3]. Half-lives of mRNAs are typically much shorter than a corresponding protein, which means that transcriptional responses to an exercise stimulus are visible in a shorter time frame, but conversely may be difficult to observe depending on the specific timing of muscle samples relative to exercise cessation [5], [9]. Whether an acutely exercise-responsive mRNA or protein is observed to *accumulate* during the course of a training period is determined principally by the half-life of the product relative to the timing of muscle biopsies. For example, exercise training increases skeletal muscle GLUT4 protein content [49], coincident with enhanced insulin sensitivity and insulin-mediated glucose disposal [50]. In the present study, a training-induced increase in GLUT4 protein occurred in the absence of an accumulation of transcript. Conversely, for other metabolic genes we observed divergent expression changes such that accumulation of both transcript and protein was observed for HKII, but not for PDK4. This is despite both genes being responsive to acute exercise [51], and instead reflects different half-lives of protein induction of the respective targets, rather than one target being less or more ‘important’ to the adaptive process.

On the morning following the first training session, the mtDNA/nDNA ratio was *reduced*. A short-term reduction in mtDNA after a single bout of exercise, an effect related to the intensity of the exercise bout, is suggested as a critical early stimulus for mitochondrial adaptation [52], [53]. In addition, a two-fold increase in protein content for a regulator of mitochondrial fission, DRP-1, occurred at the same time-point, but returned to basal levels before being elevated later in the training period. This first bout response is consistent with proposal that many acutely responsive genes are part of a generalized stress response that is triggered in “uninitiated” skeletal muscle before a more specific response to training is fine-tuned [54]. In fact, microarray data from acute exercise vs. exercise training often demonstrates little direct connection between acute upregulation and chronic accumulation of transcripts [55], [56]. However, because even in well-trained skeletal muscle, acute exercise can transiently increase metabolic and mitochondrial gene expression [7], it is likely that genes that demonstrate responsiveness to exercise training are causally-related to recovery and adaptation processes. Our findings support the proposal that transcriptional ‘capacity’ in human skeletal muscle is extremely sensitive to the onset of exercise training [10], [57].

### Perspectives and Summary

Historically, skeletal muscle biopsies are taken some time after the cessation of training, e.g. 48–96 h, to avoid the acute effects of the last exercise bout. An end-point study design provides limited temporal resolution such that transient responses in certain genes may be missed, whereas conclusions may be limited in the absence of time course data. As gene expression can be controlled at various points beyond transcription, the extent to which a protein changes in response to an adaptive stimulus such as exercise cannot be predicted from alterations in mRNA expression. Moreover, associated phenotypic manifestations do not take place until a change in the concentration or activity of the encoded protein occurs. Given the rapid kinetics of transcriptional activation, mRNA stability and protein half-lives [3], our approach, and recently that of others [10], was to employ multiple muscle biopsies to improve the temporal resolution regarding the molecular responses to frequently-repeated exercise. To this end, the link between repeated *transient* pulses of mRNA leading to an increase in protein product was recently reported during two weeks of HIIT [10]. We observed broadly similar results in terms of time course and magnitude of mRNA and protein induction for factors including PGC-1α, COXIV and CS. However, whereas we did not observe an increase in mtDNA/nDNA ratio, an increase in mtDNA/nDNA ratio was observed in response to HIIT, coincident with increased protein expression of regulators of mitochondrial biogenesis such as NRF-2, Mfn-1 and the fission proteins DRP-1 and Fis-1. We speculate that the despite the greater overall exercise *duration* in our study, the higher *intensity* of training during HIIT (reviewed in [58]) may have provided a greater stimulus for mitochondrial adaptation as classically described [59].

In summary, adaptations to chronic exercise training are well-established [1], but few studies have focused comprehensively on the transcriptional regulation of these adaptations. We demonstrate divergent effects of exercise training on mRNA and protein expression for a range of transcriptional regulators and their metabolic and mitochondrial targets in skeletal muscle of previously untrained men. Thus, the regulation of gene expression by exercise is demonstrated on a time course- and gene-specific basis, and is extremely sensitive to the initial changes in contractile activity. Overall, these data are consistent with the paradigm that initial signaling events associated with acute exercise induce pulsed, transient alterations in mRNA expression that eventually lead to a same-directional change in protein content and enzyme activity after a period of exercise training.

## Supporting Information

File S1 **Includes Tables S1 and S2. Table S1. List of targets for expression analysis in human skeletal muscle in response to fourteen consecutive days of aerobic exercise training. Table S2. Training log describing energy expenditure and relative intensity of the training programme.**
(PDF)
