# Supplementary material for: Time Course Analysis Reveals Gene-Specific Transcript and Protein Kinetics of Adaptation to Short-Term Aerobic Exercise Training in Human Skeletal Muscle
Source: PLoS One. 2013 Sep 12;8(9):e74098. doi: 10.1371/journal.pone.0074098 (PMC3771935; doi:10.1371/journal.pone.0074098)
Supplement: File S1 — Includes Tables S1 and S2. Table S1. List of targets for expression analysis in human skeletal muscle in response to fourteen consecutive days of aerobic exercise training. Table S2. Training log describing energy expenditure and relative intensity of the training programme. (PDF) [file pone.0074098.s001.pdf]

{SUPPORTING INFORMATION}

Table S1. List of targets for expression analysis in human skeletal muscle in response to fourteen consecutive days of endurance exercise training.

Target	Abbreviation	Gene symbol (HGNC)	EntrezGene ID	Assay ID	UniProt/SwissKB ID	Primary antibody	Catalogue number
Cytochrome c oxidase subunit IV isoform 1	COXIV	*COX4I1*	1327	Hs00266371_m1	P13073	Cell Signaling	#4844
Carnitine palmitoyltransferase 1B	CPT1	*CPT1B*	1375	Hs00189258_m1	Q92523	Santa Cruz	sc-20670
Cyclic-AMP response element binding protein	CREB	*CREB1*	1385	Hs00231713_m1	P16220	Cell Signaling	#9197
Citrate synthase	CS	*CS*	1431	Hs02574374_s1	O75390	Proteintech	16131-1-AP
Cytochrome c	Cyt c	*CYCS*	54205	Hs01588973_m1	P99999	BD Pharmingen	556443
Dynamin-related protein 1	DRP-1	*DNM1L*	10059	Hs00247147_m1	O00429	Santa Cruz	sc-32898
Estrogen-related receptor α	ERRα	*ESRRA*	2101	Hs01067166_g1	Q569H8	Abcam	ab76228
Forkhead box O1	FOXO1	*FOXO1*	2308	Hs00231106_m1	Q12778	Cell Signaling	#9454
Facilitated glucose transporter, member 4	GLUT4	*SLC2A4*	6517	Hs00168966_m1	P14672	Chemicon	ab1346
Hexokinase II	HKII	*HK2*	3099	Hs00606086_m1	P52789	Cell Signaling	#2106
Mitofusin 1	Mfn1	*MFN1*	55669	Hs00966851_m1	Q8IWA4	N/A	
Mitofusin 2	Mfn2	*MFN2*	9927	Hs00208382_m1	O95140	Santa Cruz	sc-50331
Nuclear respiratory factor 1	NRF-1	*NRF1*	4899	Hs00192316_m1	Q16656	N/A	
Nuclear respiratory factor 2	NRF-2	*GABPA*	2551	Hs00745591_s1	Q06546	Abcam	ab22838
Pyruvate dehydrogenase kinase 4	PDK4	*PDK4*	5166	Hs00176875_m1	Q16654	Abcam	ab63157
PPARγ coactivator 1 α	PGC-1α	*PPARGC1A*	10891	Hs00173304_m1	Q9UBK2	Calbiochem	ST1202
PPARγ coactivator 1β	PGC-1β	*PPARGC1B*	133522	Hs00370186_m1	Q86YN6	N/A	
Peroxisome proliferator-activated receptor (PPAR) δ	PPARδ	*PPARD*	5467	Hs00602622_m1	Q03181	N/A	
PGC-1-related coactivator	PRC	*PPRC1*	23082	Hs00209379_m1	Q5VV67	N/A	
Nuclear receptor interacting protein, 140 kDa	RIP140	*NRIP1*	8204	Hs00534035_s1	P48552	Abcam	ab3425
Mitochondrial transcription factor A	Tfam	*TFAM*	7019	Hs01082775_m1	Q00059	Abcam	ab47517

Relative mRNA abundance was determined by qPCR using Assay-On-Demand® primer pairs and probes (P/N 4331182, Taqman® Gene Expression Assays, Applied Biosystems) for the above listed gene targets. Primary antibodies directed against protein targets, where available, as reported in the text are listed above. HGNC, Human Gene Organisation (HUGO) Gene Nomenclature Committee; Abcam, Cambridge, UK; BD Pharmingen, Franklin Lakes, NJ, USA; Bethyl Laboratories, Montgomery, TX, USA; Calbiochem, now trading as Millipore, Billerica, MA, USA; Cell Signaling, Beverly, MA, USA; Chemicon, now trading as Millipore; Santa Cruz Biotechnology, Santa Cruz, CA, USA; Proteintech Group, Chicago, IL, USA; Upstate, now trading as Millipore.

{SUPPORTING INFORMATION}

Table S2. Training log describing energy expenditure and relative intensity of the training programme.

	Sessions 1-7	Sessions 8-14	Sessions 1-14
**VO_2 (L min^{-1})**	2.19 ± 0.10	2.32 ± 0.12	2.26 ± 0.11
**EE (kcal)**	658 ± 30	697 ± 37	677 ± 33
**% PRE VO_{2peak}**	78.2 ± 1.7	82.7 ± 2.0	80.5 ± 1.9
**% POST VO_{2peak}**	66.9 ± 2.0	70.6 ± 2.6	68.8 ± 2.3

Average oxygen uptake (VO_2), energy expenditure (EE) and relative intensity (%VO_{2peak} of either pre- or post-training aerobic capacity) of first seven (sessions 1-7), second seven (session 8-14) and all fourteen (sessions 1-14) training sessions during the exercise training programme. Values are mean ± SEM, n=8.
